# Complication, fusion, and revision rate in the lumbar cortical bone trajectory and pedicle screw fixation techniques: a systematic review and meta-analysis

**DOI:** 10.1186/s13018-023-03820-7

**Published:** 2023-05-25

**Authors:** Yixi Wang, Alafate Kahaer, Abulikemu Maimaiti, Hailong Guo, Paerhati Rexiti

**Affiliations:** 1 First Clinical Medical College, Xinjiang Medical University, Urumqi, China; 2 Department of Spine Surgery, The First Affiliated Hospital of Xinjiang Medical University, Urumqi, China; 3 Key Laboratory of High Incidence Disease Research in Xingjiang (Xinjiang Medical University), China Ministry of Education, Urumqi, China; 4 Xinjiang Clinical Research Center for Orthopedics, Xinjiang Medical University, Urumqi, China

**Keywords:** Cortical bone trajectory, Pedicle screw, Complication rate, Fusion rate, Revision rate, Single-arm meta-analysis

## Abstract

**Background:**

To obtain the complication rate, fusion rate, and revision rate of the lumbar cortical bone trajectory technique and pedicle screw fixation technique in lumbar interbody fusion surgery by single-arm meta-analysis and lay a basis for orthopedic surgeons to select the fixation techniques and perioperative management.

**Methods:**

PubMed, Ovid Medline, Web of Science, CNKI, and Wanfang databases were searched comprehensively. Data extraction, content analysis, and quality assessment of the literature were performed by two independent reviewers according to the Cochrane Collaboration guidelines using R and STATA software for single-arm meta-analysis.

**Results:**

The total complication rate of the lumbar cortical bone trajectory technique was 6%, including a hardware complication rate of 2%, ASD (adjacent segment degeneration) rate of 1%, wound infection rate of 1%, dural damage rate of 1%, hematoma rate tending to 0%, fusion rate of 94%, and revision rate of 1%. Lumbar pedicle screw fixation techniques had a total complication rate of 9%, with a hardware complication rate of 2%, ASD rate of 3%, wound infection rate of 2%, dural damage rate of 1%, hematoma rate tending to 0%, fusion rate of 94%, and revision rate of 5%. This study was registered with PROSPERO, CRD42022354550.

**Conclusion:**

Lumbar cortical bone trajectory was associated with a lower total complication rate, ASD rate, wound infection rate, and revision rate than pedicle screw fixation. The cortical bone trajectory technique reduces the incidence of intraoperative and postoperative complications and can be an alternative in lumbar interbody fusion surgery.

**Supplementary Information:**

The online version contains supplementary material available at 10.1186/s13018-023-03820-7.

## Introduction

In 1959, the pedicle screw (PS) fixation technique that can simultaneously penetrate the three-column structure of the spine was proposed [1, 2]. However, pedicle screws used in patients with osteoporosis are prone to loosening and breakage due to the destruction of the trabecular structure and loss of bone mass resulting in reduced holding strength of the screw [3–5]. Santoni et al. proposed the cortical bone trajectory (CBT) technique in 2009 and compared with the pedicle screw (PS) fixation technique, the special screw trajectory of CBT allows most of the screw surrounded by the cortical bone, giving it better mechanical stability and fixation strength [6–10]. In recent years, the CBT technique has gradually gained clinical favor by its advantages of smaller wounds, shorter operative time, and less intraoperative blood loss [11]. However, there was no study that discussed the specific complication, fusion, and revision rate of the two techniques in detail. A single-arm meta-analysis was then performed to provide a reference for the selection of fixation techniques in lumbar interbody fusion surgery and perioperative management.

## Methods

### Literature search

**Fig. 1:**
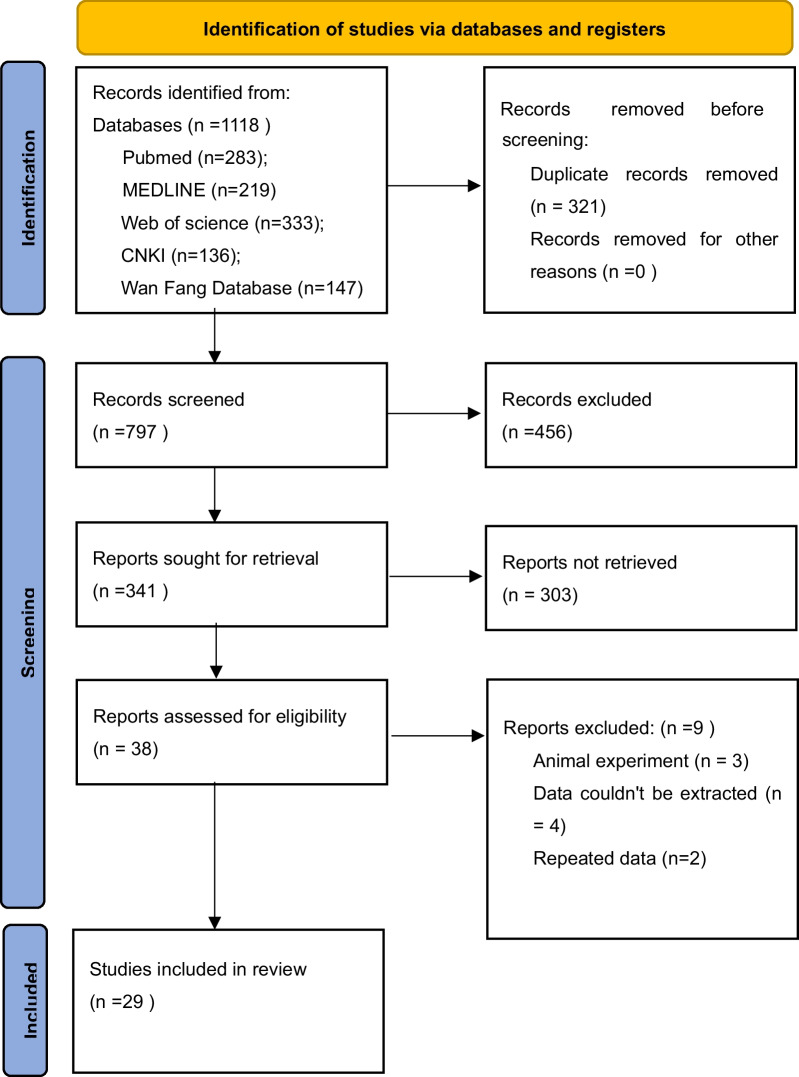
Flow diagram of study selection [12]

PubMed, Ovid Medline, Web of Science, CNKI, and Wanfang databases were searched for papers published until July 2022 using the following strategies: “Lumbar,” “Pedicle screw,” “PS,” “Traditional trajectory,” “TT,” “Cortical bone trajectory,” “cortical bone trajectory screw,” “Cortical bone screw,” “Cortical screw,” “CS,” “CBS,” “CBT,” “CBTS” with various combinations of the “AND” and “OR.” The references of all retrieved literature were manually searched one by one to improve the recall rate of the literature, and the language was limited to English and Chinese. The systematic review and single-arm meta-analysis were performed with the Preferred Reporting Items for Systematic Reviews and Meta-Analyses (PRISMA) statement and A Measurement Tool to Assess Systematic Reviews 2 (AMSTAR2) [12]. A flow diagram of the literature searching strategy is shown in Fig. 1.

### Study selection and data extraction

After completion of the literature search, all retrieved studies were checked and duplicates were removed. Two reviewers (Wang and Kahaer) independently assessed the quality of each retrieved literature to determine whether they were included and then cross-checked, with a third evaluator (Rexiti) handling any disagreements. After selection, two independent reviewers (Wang and Kahaer) extracted baseline data from the included literature, including authors, publication date, study type, number of events, mean age, gender, follow-up time, and fusion technique. Then, the type and number of complications, number of patients with bone fusion, and number of patients with revision in each literature were extracted to the predesigned data extraction sheet. This study was registered with PROSPERO and updated regularly, ID CRD42022354550.

### Inclusion and exclusion criteria

Inclusion criteria were as follows: (1) Literature included patients with lumbar interbody fusion surgery using CBT screw and PS; (2) Preoperative diagnosis of lumbar degenerative diseases, lumbar tuberculosis; (3) Surgical levels were single or double; and (4) Literature reported one of the rates of complication, fusion, and revision.

Exclusion criteria were as follows: (1) Only include the CBT or PS in lumbar interbody fusion surgery; (2) Previous history of lumbar surgery; (3) Patients with lumbar tumors and fractures; (4) Severe medical system diseases, such as chronic obstructive pulmonary disease, coagulation abnormalities, long-term use of glucocorticoids, hypertension in grade III and above; (5) Three or more surgical segments; (6) Follow-up less than 6 months (7) Letter to editor, review, and conference paper.

### Quality assessment

Randomized controlled trials (RCTs) were assessed using the Cochrane Risk Bias Assessment Tool, and non-randomized controlled studies (cohort studies, case–control studies) were assessed using the Newcastle–Ottawa Scale (NOS).

### Statistical analysis

Statistical methods were described in PRISMA statement. Forest plots were drawn by R software version 4.2.1. After importing the raw data into R software, PRAW, PLOGIT, PLN, PAS, and PFT were used to transform the original rates of complication, fusion, and revision of each literature, respectively. The transformed rate was tested for normal distribution. The method closest to normal distribution was selected according to the test results. Then, the combined rate and 95% confidence interval (CI) were obtained by metaprop function and a forest plot was drawn. When *I*^2^ < 50% and/or *P* > 0.1 (low heterogeneity), a fixed-effect model was selected; otherwise, the random-effect model was selected. STATA 16 (Stata Corp., College Station, TX, USA) was used to analyze the sensitivity when *I*^2^ > 50%. The specific method was to exclude the literature one by one to obtain the combined conversion rate and 95% CI for determining the effect of each literature on the combined effect size.

## Results

### Literature characteristic and quality assessment

The literature search yielded 1118 studies, including 835 in English and 283 in Chinese. After removing duplicates, 797 studies were retrieved. Screening by title and abstract left 341 studies for full-text analysis. After full-text screening, it left 38 studies. According to the inclusion and exclusion criteria, 29 studies [13–41] met the inclusion criteria, 22 in English and 6 in Chinese. Two RCTs [32, 34], six prospective studies [13, 14, 20, 27, 35, 36], and 21 retrospective studies [15–19, 21–26, 28–31, 33, 37, 38, 40, 41] were included. A total of 982 patients with CBT and 1105 patients with PS were compared. One retrospective cohort study [38] did not report the specific follow-up time, but the hardware complication (intraoperative screw malposition) did not require long-term follow-up;thus, it was included. Among the included studies, Lai et al. [26] included patients with lumbar tuberculosis and the rest research only included patients with lumbar degenerative diseases. Summary of study characteristics is presented in Table 1. Although the follow-up population of Lai’s study was osteoporotic patients with lumbar spinal tuberculosis, we only cited the occurrence of Hardware events in that study, so the impact on this study was not significant.

**Table 1:** Study characteristics

Included studies	Study design	Country	Fusion technique	Patients number		Mean age		Gender (M/F)		Follow-up (months)	
				CBT	PS	CBT	PS	CBT	PS	CBT	PS
Marengo et al. 2018 [13]	Prospective cohort	Italy	PLIF	20	20	45.75 ± 9.63	54 ± 12.01	12/8	9/11	12	12
Sakaura et al. 2019 [14]	Prospective cohort	Japan	PLIF	102	77	67.5 ± 9.2	66.4 ± 10.5	35/67	28/49	36	36
Sakaura et al. 2021 [15]	Retrospective cohort	Japan	PLIF	24	37	67.5 ± 9.2	69.3 ± 10.0	19/18	10/14	24	24
Takenaka et al. 2017 [16]	Retrospective cohort	Japan	PLIF	42	77	65.8 ± 8.1	66.0 ± 11.2	18/24	31/46	12	12
Kasukawa et al. 2015 [17]	Retrospective cohort	Japan	TLIF	10	10	67	63	3/7	6/4	8	16
Konomi et al. 2020 [18]	Retrospective cohort	Japan	PLIF	30	37	NA	NA	NA	NA	24	24
Ninomiya et al. 2016 [19]	Retrospective cohort	Japan	PLIF	11	10	62.2 ± 2.5	61.4 ± 2.6	7/4	5/5	12	12
Orita et al. 2015 [20]	Prospective cohort	Japan	TLIF	20	20	63.5 ± 9.4	63.7 ± 14.3	11/9	12/8	12	12
Nakajima et al. 2020 [21]	Retrospective cohort	Japan	PLIF	36	68	69.7 ± 10.0	69.1 ± 10.8	16/20	25/43	12	12
Sakaura et al. 2016 [22]	Retrospective cohort	Japan	PLIF	95	82	68.7 ± 9.5	67 ± 8.7	46/49	36/46	35	40
Sakaura et al. 2018 [23]	Retrospective cohort	Japan	PLIF	22	20	70.7 ± 7.3	68.3 ± 9.5	4/18	6/14	39	35
Huang et al. 2016 [24]	Retrospective cohort	China	PLIF	16	16	60.37 ± 11.07	64.12 ± 5.79	5/11	6/10	18	18
Wang et al. 2018 [25]	Retrospective cohort	China	PLIF	51	46	62.8 ± 8.7	61.9 ± 11.3	23/28	18/28	12	12
Lai et al. 2020 [26]	Retrospective case–control	China	NA	21	21	72.52 ± 9.25	71.42 ± 9.81	12/9	11/10	15	15
Liu et al. 2019 [27]	Prospective cohort	China	PLIF	50	54	68 ± 5	67 ± 5	26/24	27/27	12	12
Liu et al. 2020 [40]	Retrospective cohort	China	PLIF	22	30	56 ± 9	60.8 ± 8.7	0/22	0/30	24	24
Karki et al. 2019 [28]	Retrospective cohort	China	TLIF (CBT) PLIF (PS)	26	45	64.8 ± 8.5	66.6 ± 6.8	5/21	20/25	12	12
Peng et al. 2017 [29]	Retrospective cohort	China	PLIF	51	46	62.8	61.9	23/28	21/25	24	24
Fu et al. 2020 [41]	Retrospective cohort	China	NA	30	35	66.2 ± 2.07	66.14 ± 2.16	19/11	23/12	> 9	> 9
Zhang et al. 2022 [30]	Retrospective cohort	China	NA	27	25	51.8 ± 9.9	52.6 ± 9.7	15/12	14/11	> 36	> 36
Zhang et al. 2022 [31]	Retrospective cohort	China	PLIF	51	60	57.08 ± 9.70	55.53 ± 10.43	18/33	23/37	34	34
Lee & Ahn 2017 [32]	Randomized controlled trail	South Korea	PLIF + PLF	35	37	32.7 ± 10.1	64.2 ± 9.3	9/13	12/19	12	12
Lee & Shin 2018 [33]	Retrospective cohort	South Korea	PLIF	22	31	51.2 ± 11.9	51.7 ± 10.4	31/4	33/4	24	24
Lee et al. 2015 [34]	Randomized controlled trail	South Korea	PLIF	38	39	51.3 ± 12.4	51.9 ± 11.7	33/5	34/5	12	12
Chen et al. 2016 [35]	Prospective cohort	USA	NA	18	15	53.39 ± 1.97	59.2 ± 3.12	11/7	2/13	15	15
Chin et al. 2017 [36]	Prospective cohort	USA	NA	30	30	48 ± 3	62 ± 3	18/12	15/15	24	24
Hoffman et al. 2019 [37]	Retrospective case control	USA	MIDLF (CBT) TLIF (PS)	25	23	53.4	48.5	16/0	16/0	52.5	52.5
Wochna et al. 2018 [38]	Retrospective cohort	USA	ORIF + MIS (PS) NA (CBT)	12	59	46.50 ± 15.13	49.24 ± 17.54	NA	NA	NA	NA
Malcolm et al. 2018 [39]	Retrospective cohort	USA	TLIF	45	35	63 ± 9	57 ± 11	20/25	7/28	12	12

M, male; F, female; CBT, cortical bone trajectory; PS, pedicle screw; PLIF, posterior lumbar interbody fusion; TLIF, transforaminal lumbar interbody fusion; PLF, posterolateral lumbar fusion; MIDLF, midline lumbar fusion

**Fig. 2:**
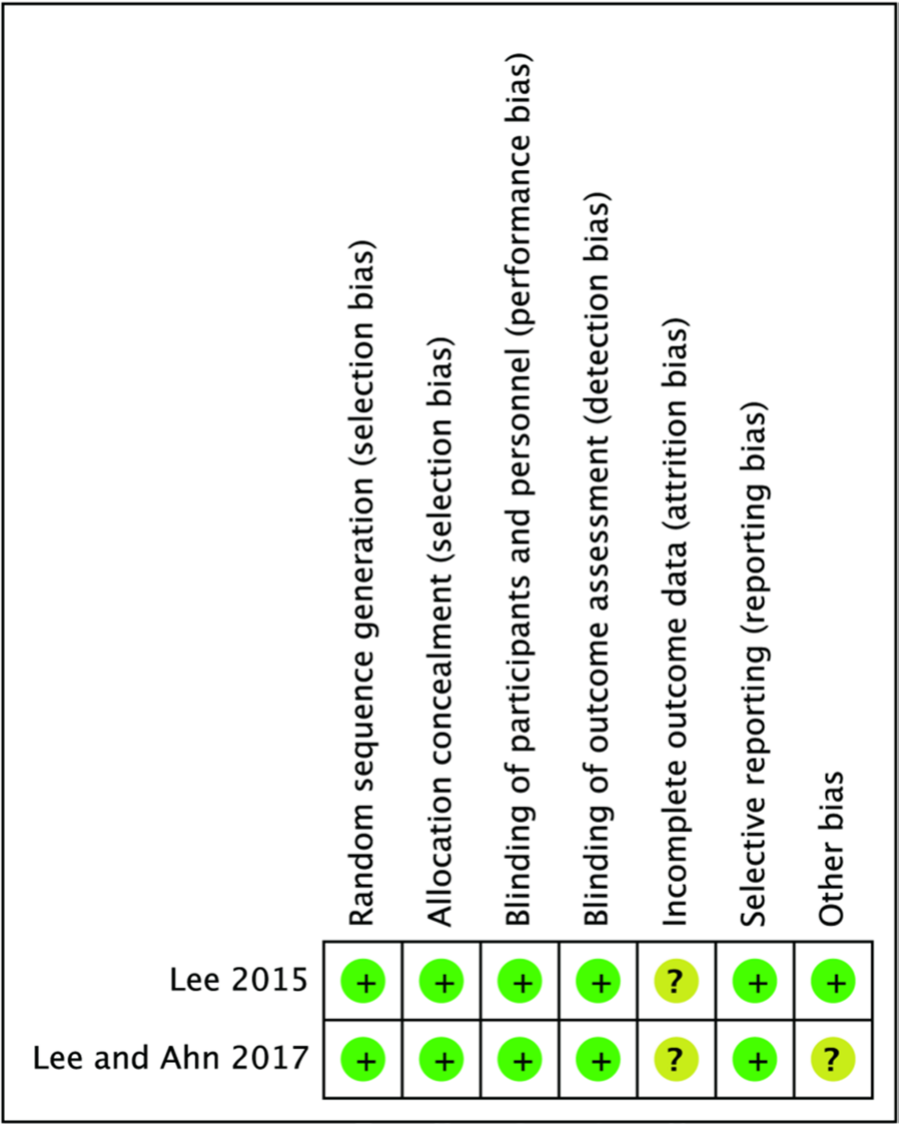
Cochrane risk bias assessment results

Two RCTs showed that the articles were of good quality, and the specific assessment result is shown in Fig. 2. The NOS assessment results showed the scores of all included studies covered high-quality 6–9 points, defined as high-quality points. The specific assessment results are shown in Table 2.

**Table 2:** Newcastle–Ottawa scale

Study	Selection	Comparability	Outcomes/exposure	Quality judgment
Marengo et al. 2018 [13]	4	2	2	8
Sakaura et al. 2019 [14]	4	2	3	9
Sakaura et al. 2021 [15]	4	2	3	9
Takenaka et al. 2017 [16]	4	2	3	9
Kasukawa et al. 2015 [17]	4	2	3	9
Konomi et al. 2020 [18]	4	2	3	9
Ninomiya et al. 2016 [19]	4	2	3	9
Orita et al. 2015 [20]	4	2	3	9
Nakajima et al. 2020 [21]	4	2	3	9
Sakaura et al. 2016 [22]	4	2	3	9
Sakaura et al. 2018 [23]	4	2	3	9
Huang et al. 2016 [24]	4	2	2	8
Wang et al. 2018 [25]	4	1	2	7
Lai et al. 2020 [26]	4	1	1	6
Liu et al. 2019 [27]	4	2	2	8
Liu et al. 2020 [40]	4	1	2	7
Karki et al. 2019 [28]	4	2	2	8
Peng et al. 2017 [29]	4	1	2	7
Fu et al. 2020 [41]	4	1	2	7
Zhang et al. 2022 [30]	4	1	3	8
Zhang et al. 2022 [31]	4	2	2	8
Lee & Shin 2018 [33]	4	2	2	8
Chen et al. 2016 [35]	4	2	3	9
Chin et al. 2017 [36]	4	1	3	8
Hoffman et al. 2019 [37]	4	2	2	8
Wochna et al. 2018 [38]	4	1	1	6
Malcolm et al. 2018 [39]	4	2	2	8

### Total complication rate

#### Total complication rate of CBT

**Fig. 3:**
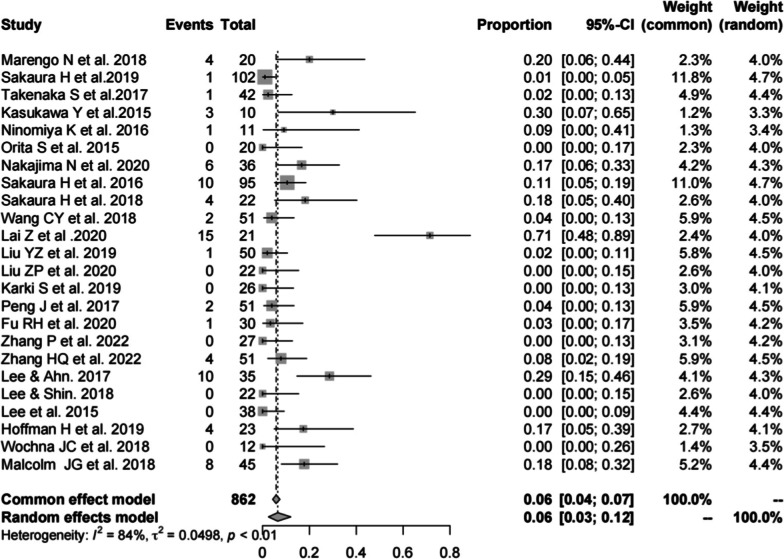
Meta-analysis of total complication rate of CBT

Twenty-four studies [13, 14, 16, 17, 19–23, 25–34, 37–41] consisting of 862 patients reported the complications of CBT (*n* = 77). There was a significant heterogeneity (*I*^2^ = 84%, *P* < 0.01). Meta-analysis was performed using a random-effects model. Combined statistics showed a total complication rate of 6% (95% CI [3, 12%]) (Fig. 3).

#### Total complication rate of PS

**Fig. 4:**
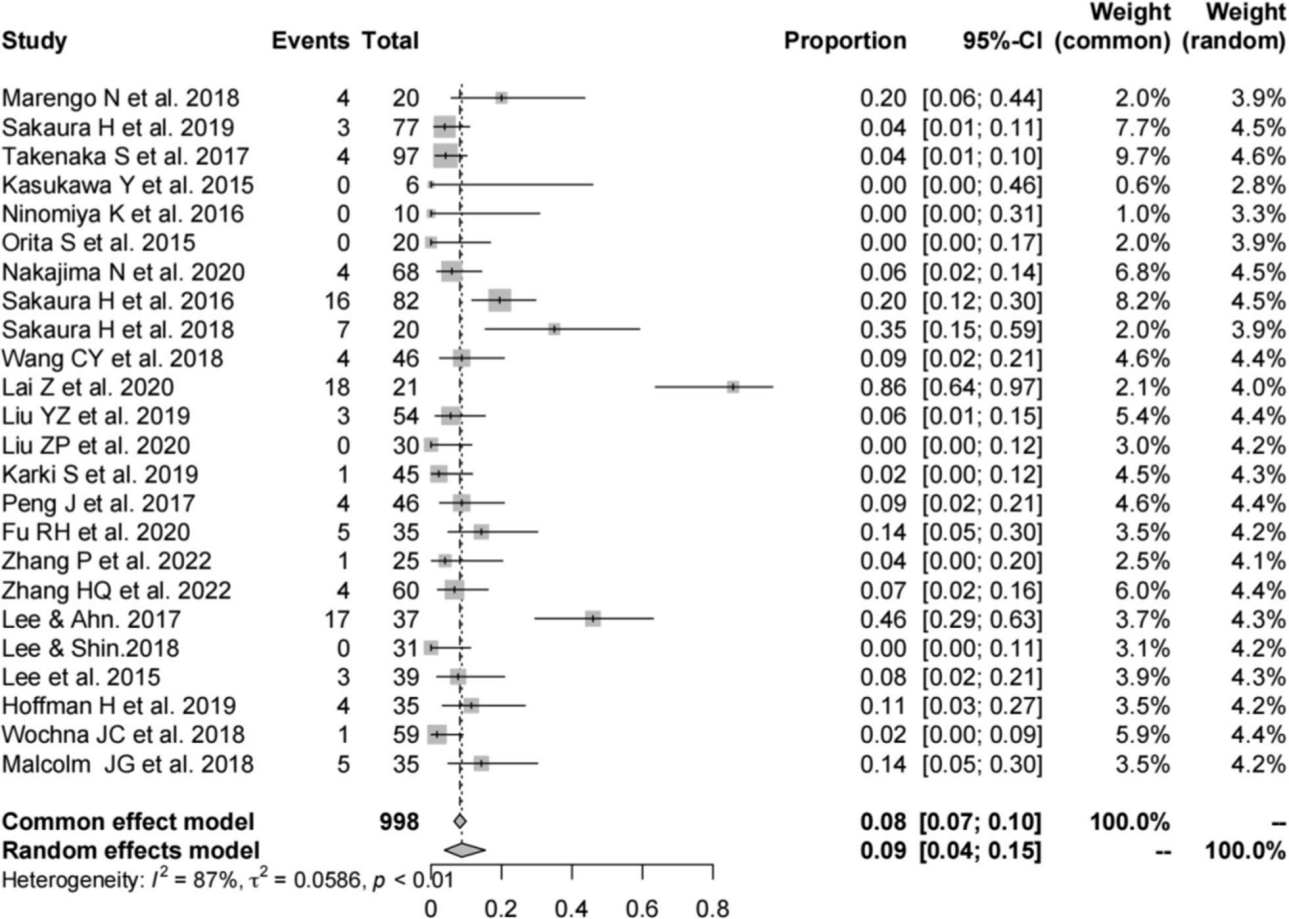
Meta-analysis of total complication rate of PS

Twenty-four studies [13, 14, 16, 17, 19–23, 25–34, 37–41] (consistent with the total complications of CBT) consisting of 998 patients reported the complications of PS (*n* = 108). There was a significant heterogeneity (*I*^2^ = 87%, *P* < 0.01). Meta-analysis was performed using a random-effects model. Combined statistics showed a total complication rate of 9% (95% CI [4, 15%]) (Fig. 4).

### Hardware complication rate

#### Hardware complications of CBT

**Fig. 5:**
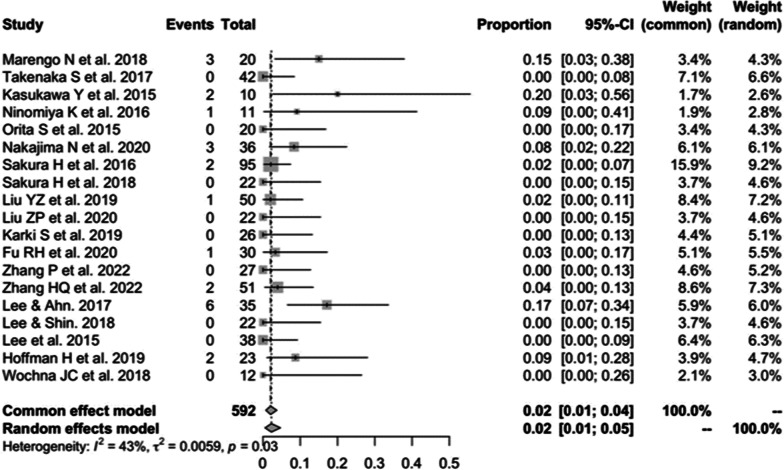
Meta-analysis of hardware complications rate of CBT

Nineteen studies [13, 16, 17, 19–23, 27, 28, 30–34, 37, 38, 40, 41] consisting of 592 patients reported the hardware complications of CBT (*n* = 23). There was no significant heterogeneity (*I*^2^ = 43%, *P* = 0.03). Meta-analysis was performed using a fixed-effects model. Combined statistics showed a hardware complication rate of 2% (95% CI [1, 4%]) (Fig. 5). Specific types of hardware complications are shown in Table 3.

**Table 3:** Types of hardware complications

Study	CBT (No.)	PS (No.)
Marengo et al. 2018	Screw malpositioning (3)	Screw malpositioning (3)
Takenaka et al. 2017	0	0
Kasukawa et al. 2015	Pedicle fracture (2)	0
Ninomiya et al. 2016	Spacer backout (1)	0
Orita et al. 2015	0	0
Nakajima et al. 2020	Pedicle fracture (3)	Pedicle fracture (2)
Sakaura et al. 2016	Screw malpositioning (2)	Screw malpositioning (3)
Sakaura et al. 2018	0	Screw malpositioning (1)
Liu et al. 2019	Fusion cage displacement (1)	Screw pullout (2)
Liu et al. 2020	0	0
Karki et al. 2019	0	0
Fu et al. 2020	Screw loosening (1)	Screw loosening (5)
Zhang et al. 2022	0	0
Zhang et al. 2022	Screw malpositioning (2)	0
Lee and Ahn 2017	Screw loosening (4) Cage subsidence (2)	Screw loosening (7) Cage subsidence (2)
Lee and Shin 2018	0	0
Lee et al. 2015	0	Screw malpositioning (2)
Hoffman et al. 2019	Screw pullout (2)	Screw pullout or loosening (2) Screw malposition (1)
Wochna et al. 2018	0	Construct failure (1)

CBT, cortical bone trajectory; PS, pedicle screw

#### Hardware complications of PS

**Fig. 6:**
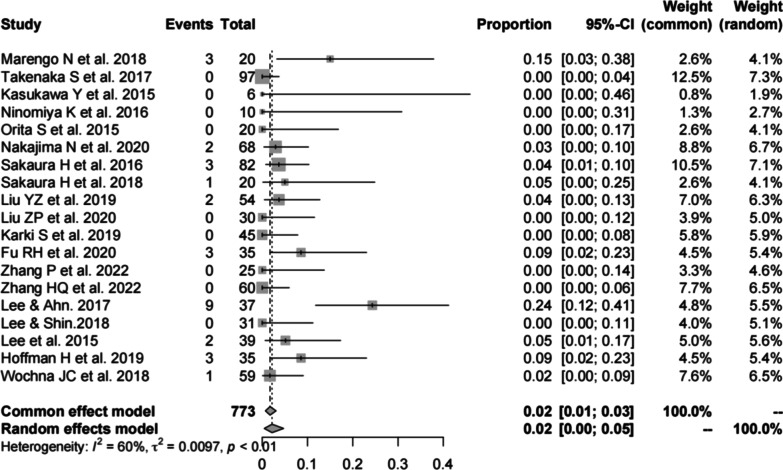
Meta-analysis of hardware complications rate of PS

Nineteen studies [13, 16, 17, 19–23, 27, 28, 30–34, 37, 38, 40, 41] (consistent with the hardware complications of CBT) consisting of 773 patients reported the hardware complications of PS (*n* = 29). There was a significant heterogeneity (*I*^2^ = 60%, *P* < 0.01). Meta-analysis was performed using a random-effects model. Combined statistics showed a hardware complication rate of 2% (95% CI [0, 5%]) (Fig. 6). Specific types of hardware complications are shown in Table 3.

### Incidence of adjacent segment degeneration (ASD)

#### ASD in CBT

**Fig. 7:**
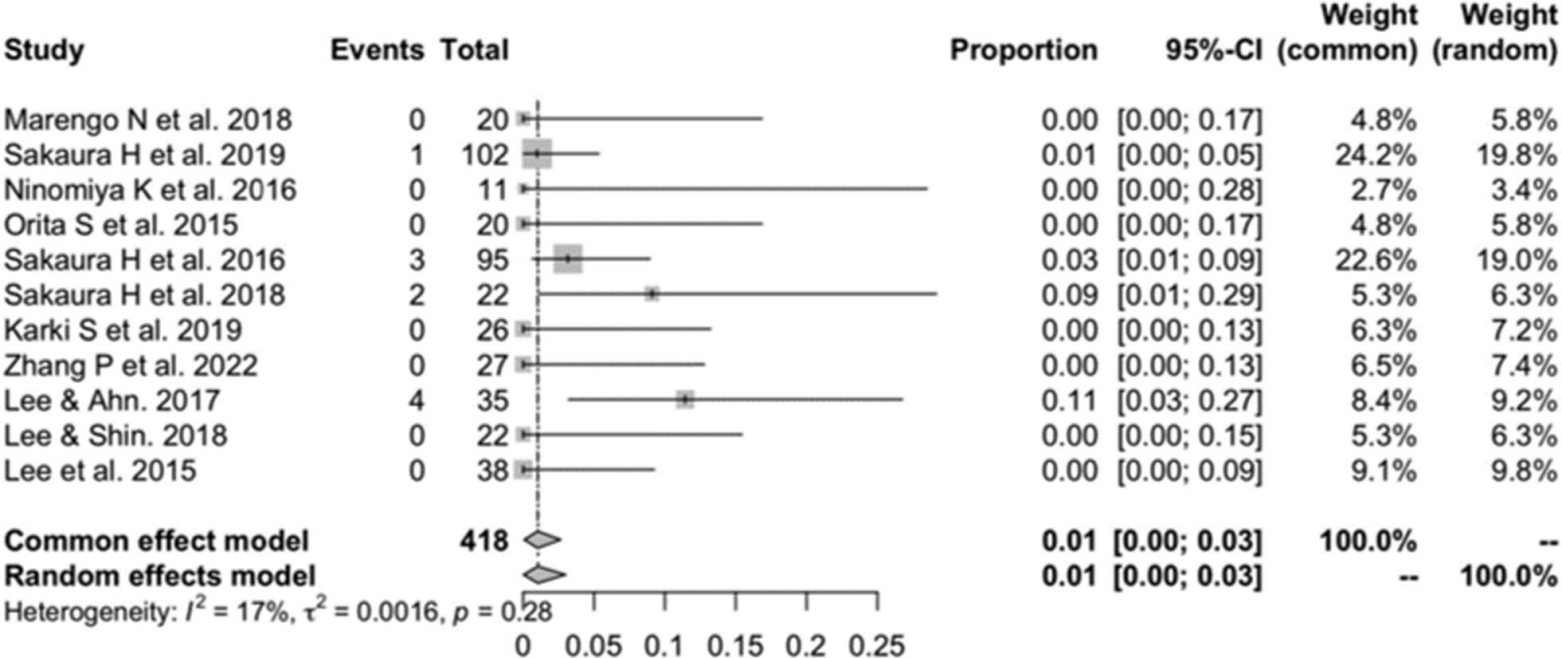
Meta-analysis of incidence of ASD in CBT

Eleven studies [13, 14, 19, 20, 22, 23, 28, 30, 32–34] consisting of 418 patients reported the incidence of ASD in CBT (*n* = 10). There was no heterogeneity (*I*^2^ = 17%, *P* = 0.28). Meta-analysis was performed using a fixed-effects model. Combined statistics showed the incidence of ASD was 1% (95% CI [0, 3%]) (Fig. 7).

#### ASD in PS

**Fig. 8:**
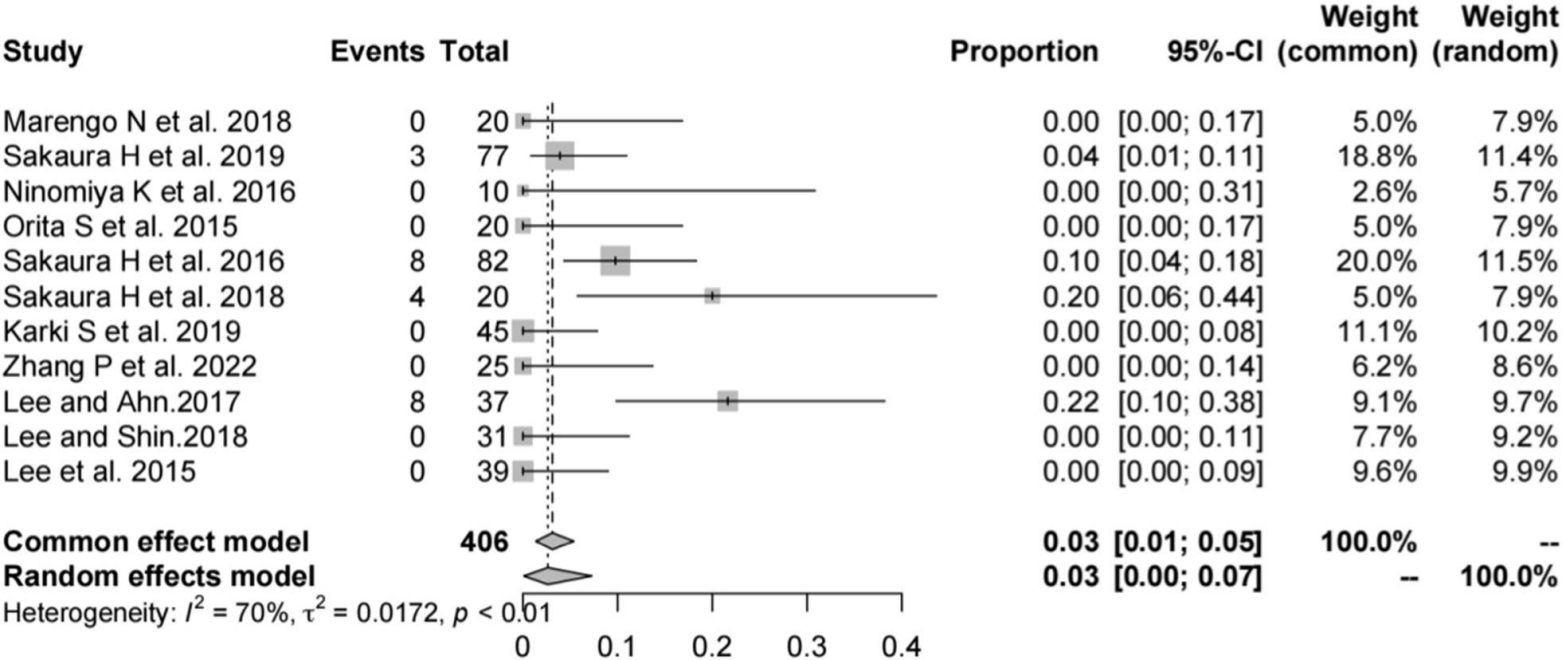
Meta-analysis of incidence of ASD in PS

Eleven studies [13, 14, 19, 20, 22, 23, 28, 30, 32–34] (consistent with the ASD in CBT) consisting of 406 patients reported the incidence of ASD in PS (*n* = 23). There was a significant heterogeneity (*I*^2^ = 70%, *P* < 0.01). Meta-analysis was performed using a random-effects model. Combined statistics showed the incidence of ASD was 3% (95% CI [0, 7%]) (Fig. 8).

### Wound infection rate

#### Wound infection of CBT

**Fig. 9:**
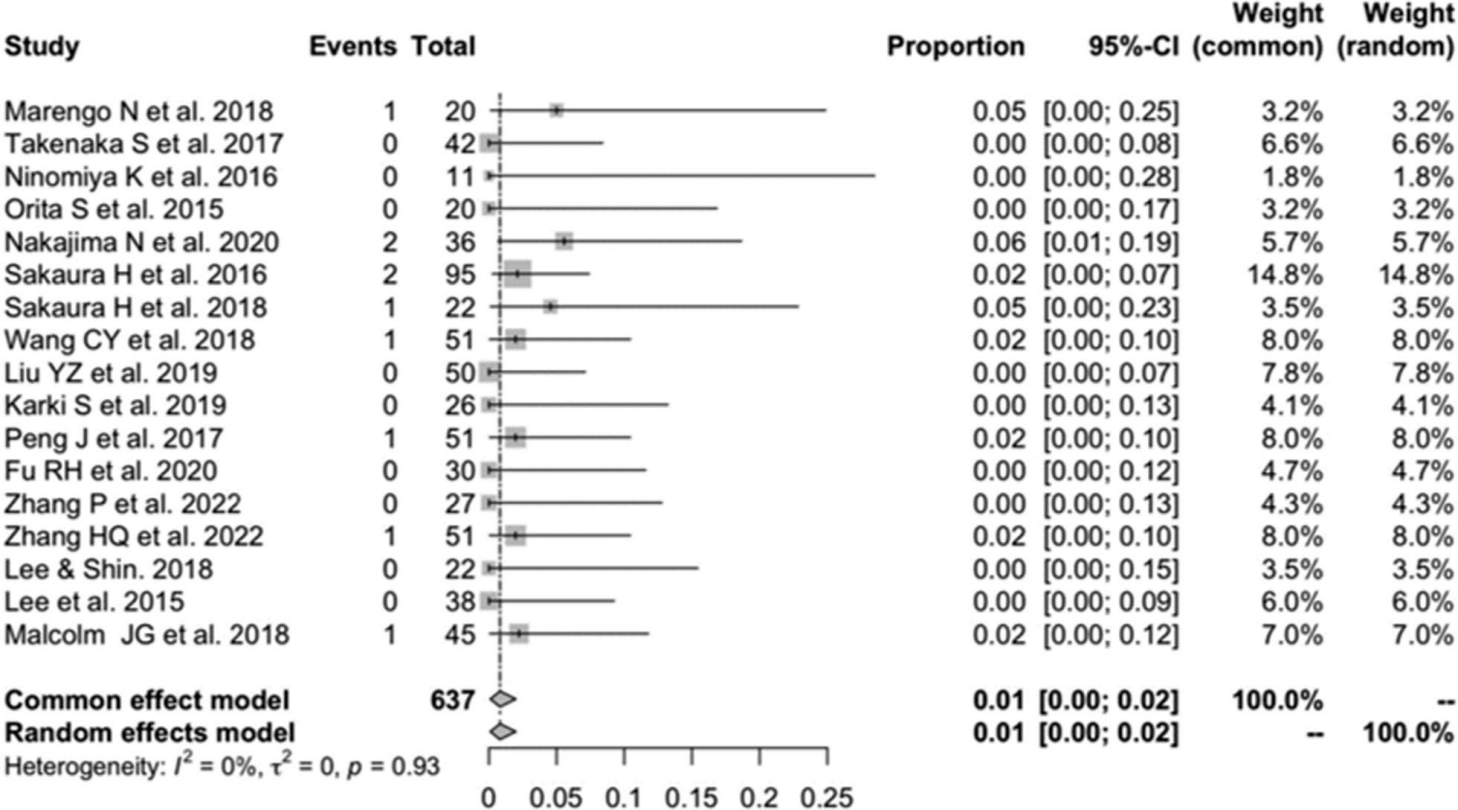
Meta-analysis of incidence of wound infection of CBT

Seventeen studies [13, 16, 19–23, 25, 27–31, 33, 34, 39, 41] consisting of 637 patients reported the wound infection of CBT (*n* = 10). There was no significant heterogeneity (*I*^2^ = 0%, *P* = 0.93). Meta-analysis was performed using a fixed-effects model. Combined statistics showed a wound infection rate of 1% (95% CI [0, 2%]) (Fig. 9).

#### Wound infection of PS

**Fig. 10:**
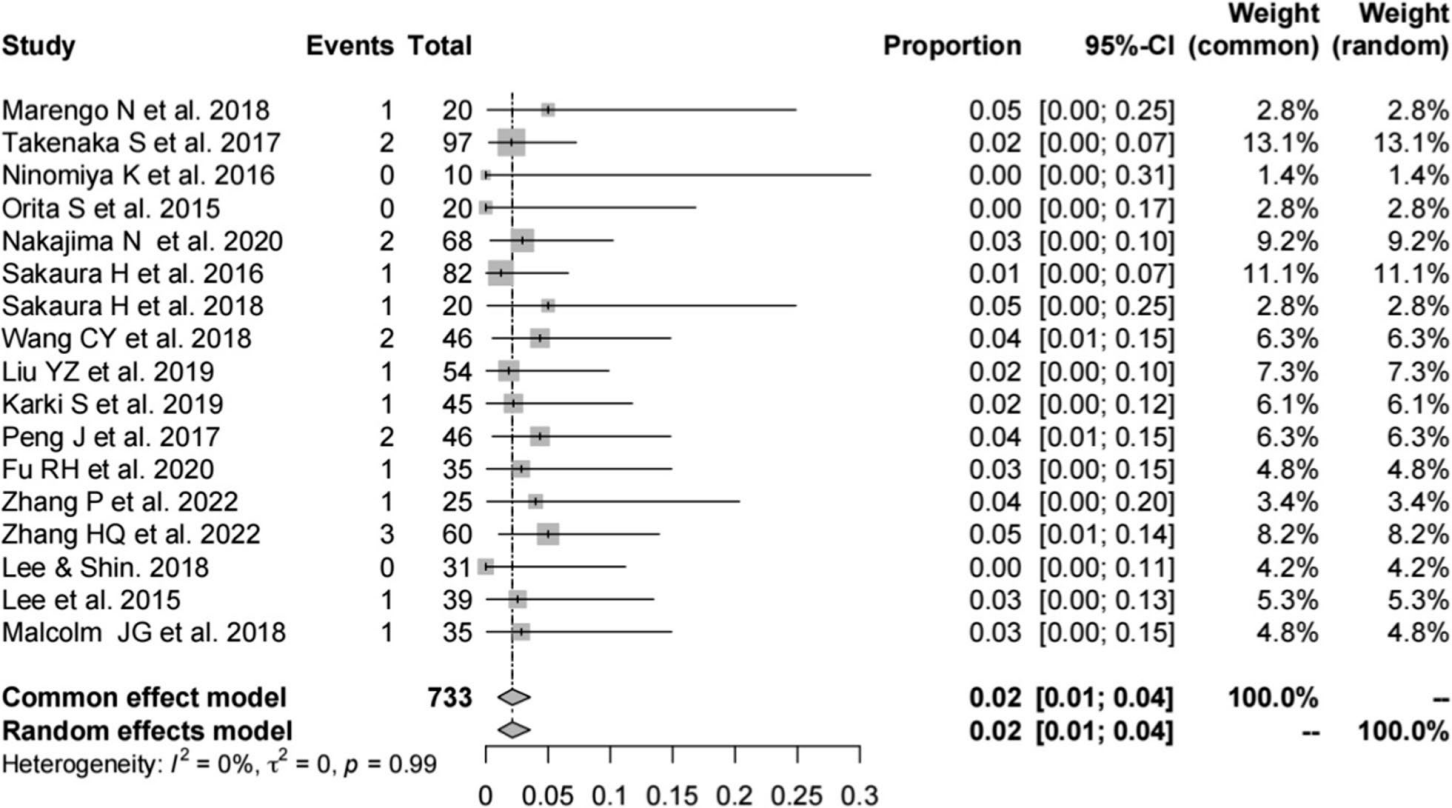
Meta-analysis of incidence of wound infection of PS

Seventeen studies [13, 16, 19–23, 25, 27–31, 33, 34, 39, 41] (consistent with wound infection of CBT) consisting of 733 patients reported the wound infection of PS (*n* = 20). There was no significant heterogeneity (*I*^2^ = 0%, *P* = 0.99). Meta-analysis was performed using a fixed-effects model. Combined statistics showed a wound infection rate of 2% (95% CI [1, 4%]) (Fig. 10).

### Incidence of dural damage

#### Incidence of dural damage in CBT

**Fig. 11:**
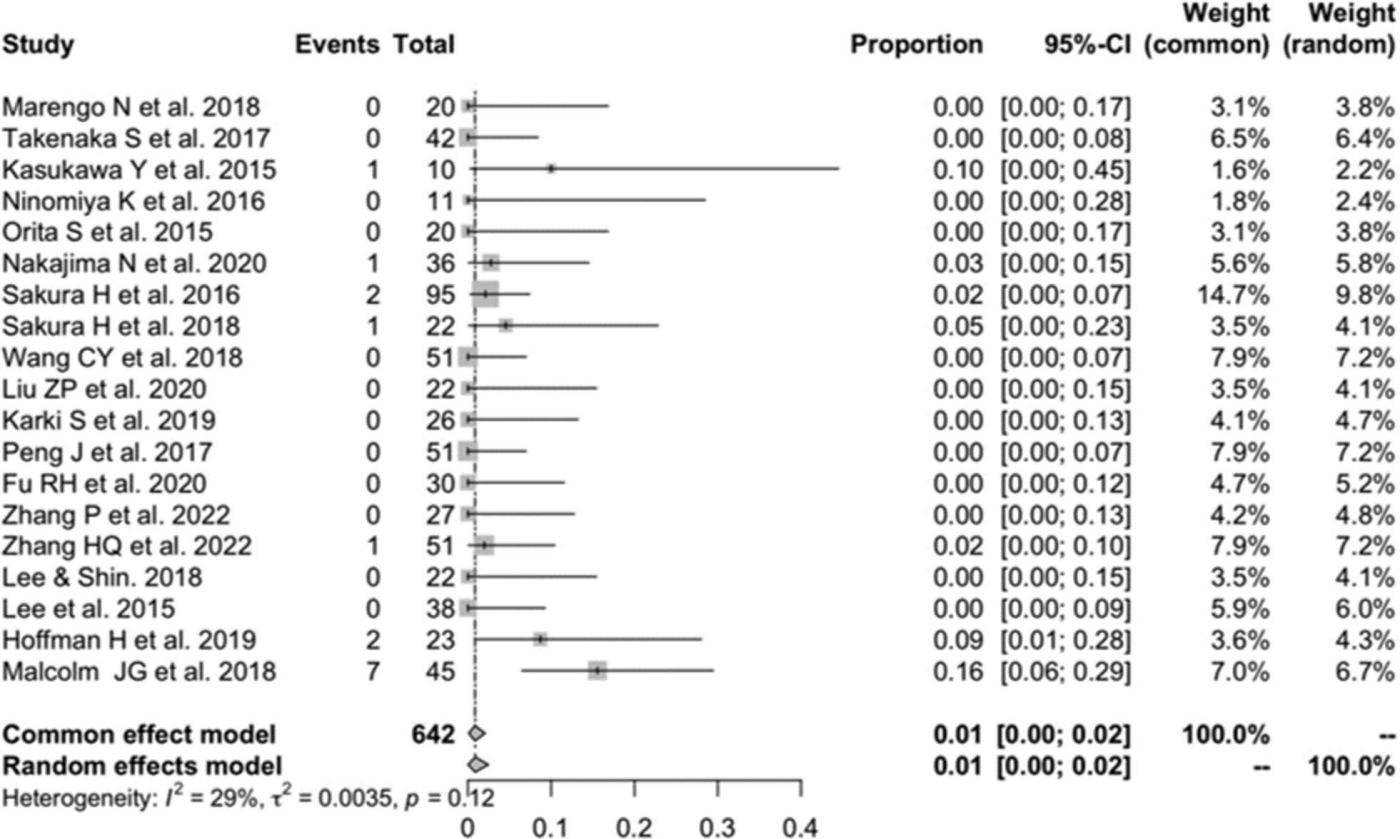
Meta-analysis of incidence of dural damage of CBT

Nineteen studies [13, 16, 17, 19–23, 25, 28–31, 33, 34, 37, 39–41] consisting of 492 patients reported incidence of dural damage in CBT (*n* = 15). There was no significant heterogeneity (*I*^2^ = 29%, *P* = 0.12). Meta-analysis was performed using a fixed-effects model. Combined statistics showed the incidence of dural damage was 1% (95% CI [0, 3%]) (Fig. 11).

#### Incidence of dural damage in PS

**Fig. 12:**
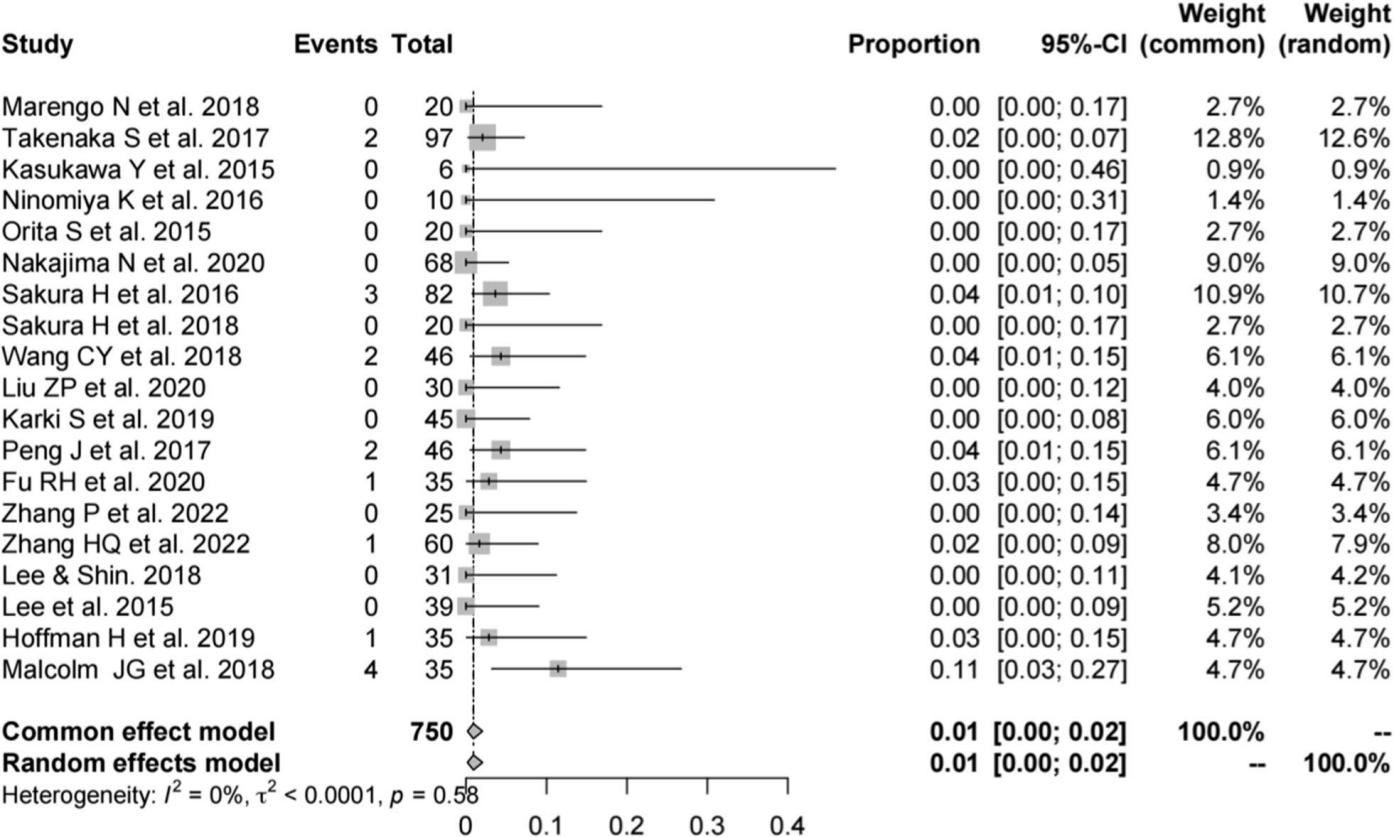
Meta-analysis of incidence of dural damage of PS

Nineteen studies [13, 16, 17, 19–23, 25, 28–31, 33, 34, 37, 39–41] (consistent with incidence of dural damage in CBT) consisting of 750 patients reported incidence of dural damage in PS (*n* = 16). There was no significant heterogeneity (*I*^2^ = 0%, *P* = 0.58). Meta-analysis was performed using a fixed-effects model. Combined statistics showed the incidence of dural damage was1% (95% CI [0, 2%]) (Fig. 12).

### Incidence of hematoma

#### Incidence of hematoma in CBT

**Fig. 13:**
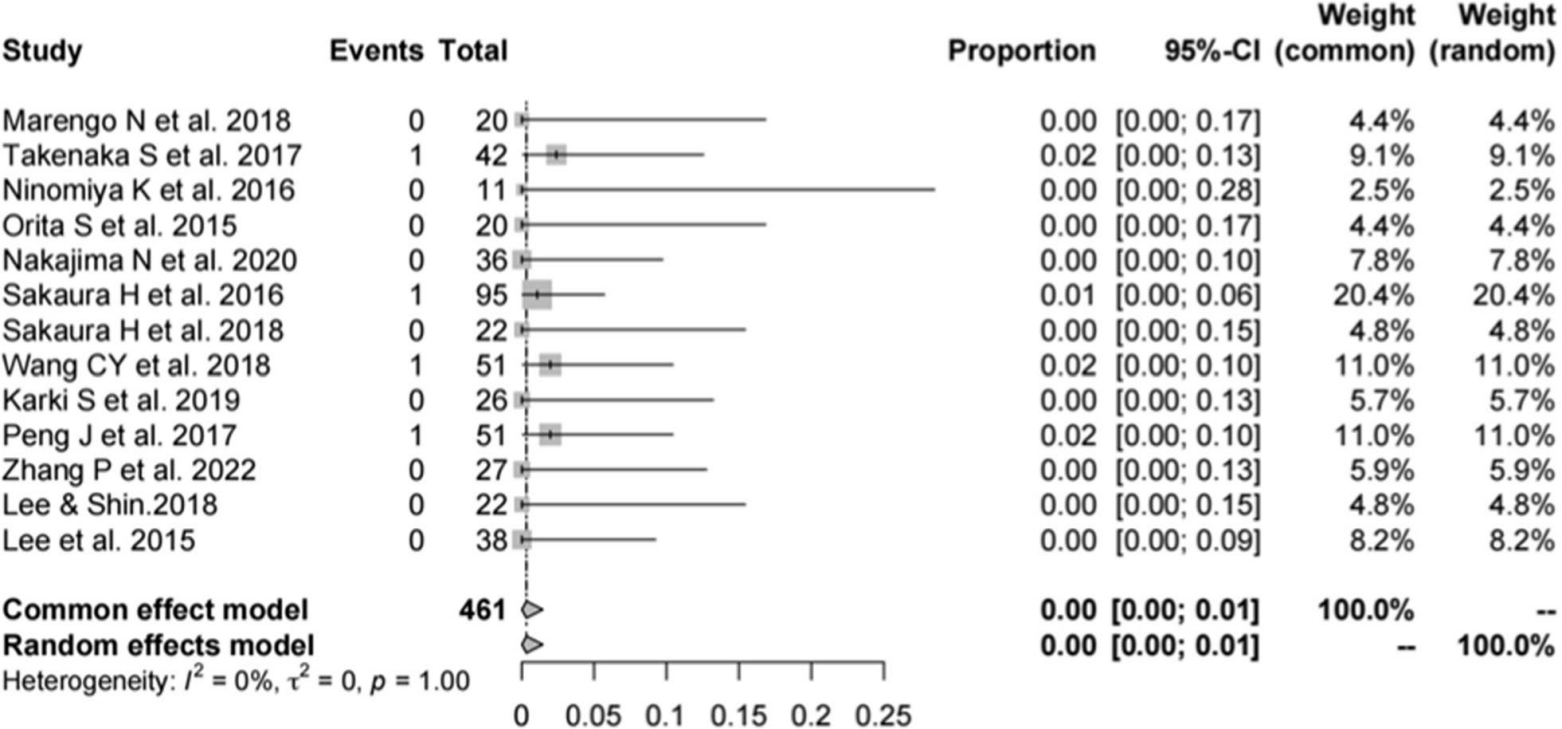
Meta-analysis of incidence of hematoma of CBT

Thirteen studies [13, 16, 19–23, 25, 28–30, 33, 34] consisting of 461 patients reported the hardware complications of CBT (*n* = 4). There was no significant heterogeneity (*I*^2^ = 0%, *P* = 1.00). Meta-analysis was performed using a fixed-effects model. Combined statistics showed the incidence of hematoma was 0% (95% CI [0, 1%]) (Fig. 13).

#### Incidence of hematoma in PS

**Fig. 14:**
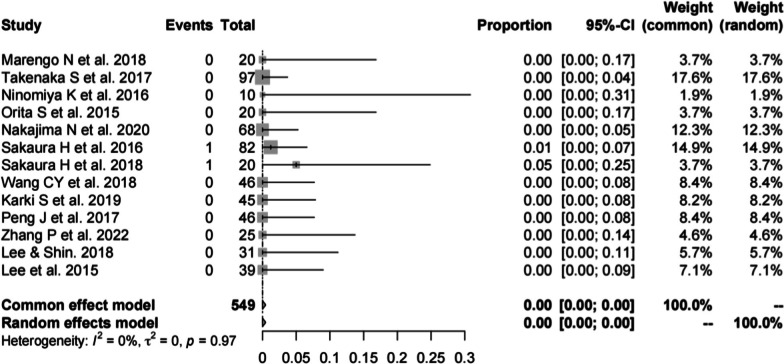
Meta-analysis of incidence of hematoma of PS

Thirteen studies [13, 16, 19–23, 25, 28–30, 33, 34] (consistent with the incidence of hematoma in CBT) consisting of 549 patients reported the hardware complications of PS (*n* = 2). There was no significant heterogeneity (*I*^2^ = 0%, *P* = 0.97). Meta-analysis was performed using a fixed-effects model. Combined statistics showed the incidence of hematoma was 0% (95% CI [0, 0%]) (Fig. 14).

### Fusion rate

#### Fusion rate of CBT

**Fig. 15:**
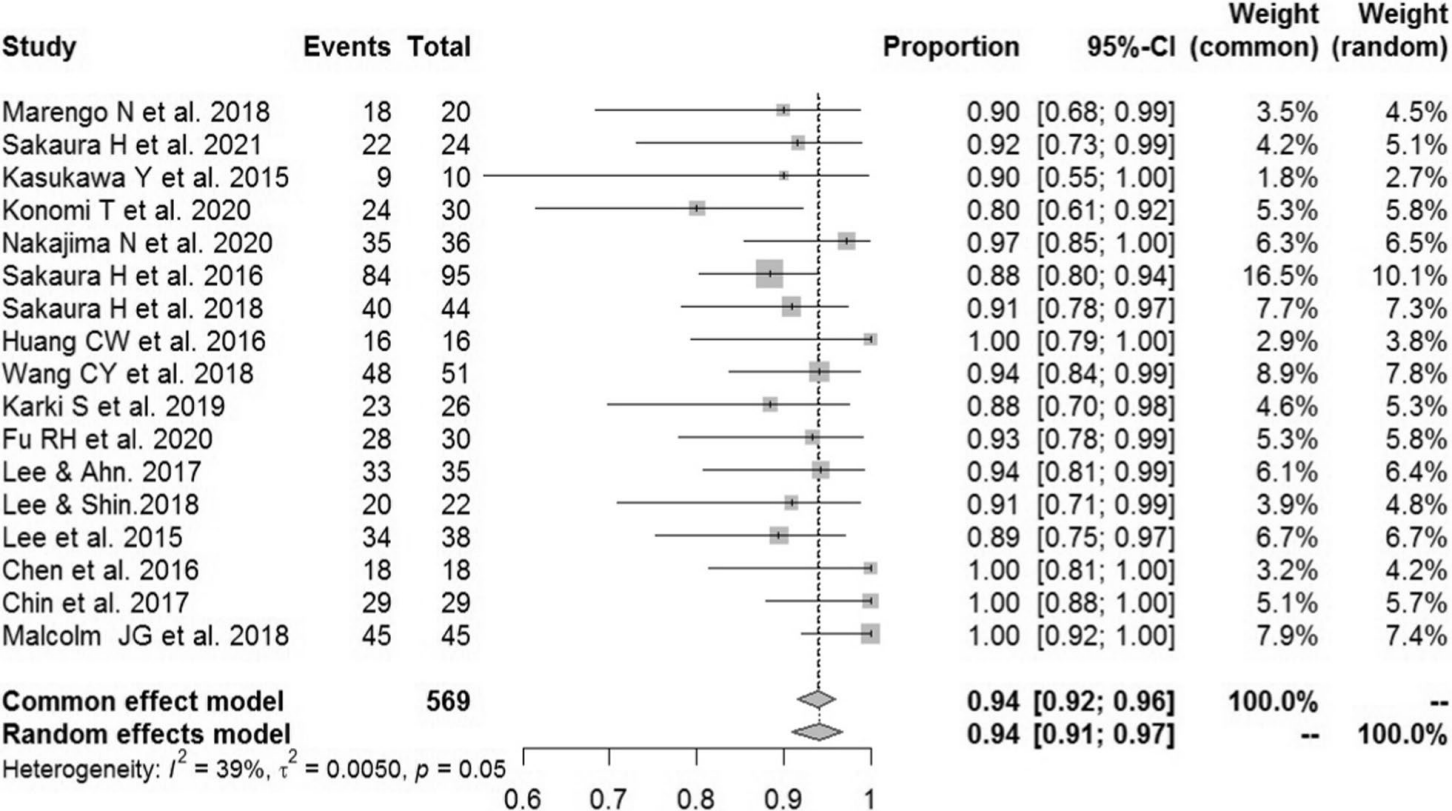
Meta-analysis of fusion rate of CBT

Seventeen studies [13, 15, 17, 18, 21–25, 28, 32–36, 39, 41] consisting of 569 patients reported the fusion rate of CBT (*n* = 526). There was no significant heterogeneity (*I*^2^ = 39%, *P* = 0.05). Meta-analysis was performed using a fixed-effects model. Combined statistics showed a fusion rate of 94% (95% CI [92, 96%]) (Fig. 15).

#### Fusion rate of PS

**Fig. 16:**
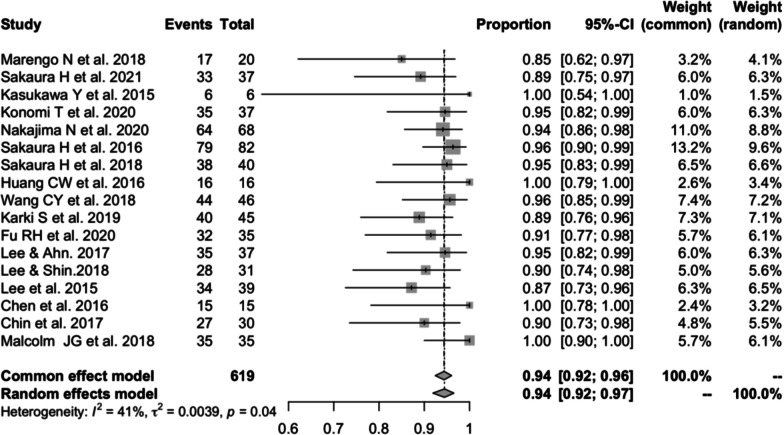
Meta-analysis of fusion rate of PS

Seventeen studies [13, 15, 17, 18, 21–25, 28, 32–36, 39, 41] (consistent with the fusion rate of CBT) consisting of 619 patients reported the fusion rate of PS (*n* = 578). There was no significant heterogeneity (*I*^2^ = 41%, *P* = 0.04). Meta-analysis was performed using a fixed-effects model. Combined statistics showed a fusion rate of 94% (95% CI [92, 96%]) (Fig. 16).

### Revision rate

#### Revision rate of CBT

**Fig. 17:**
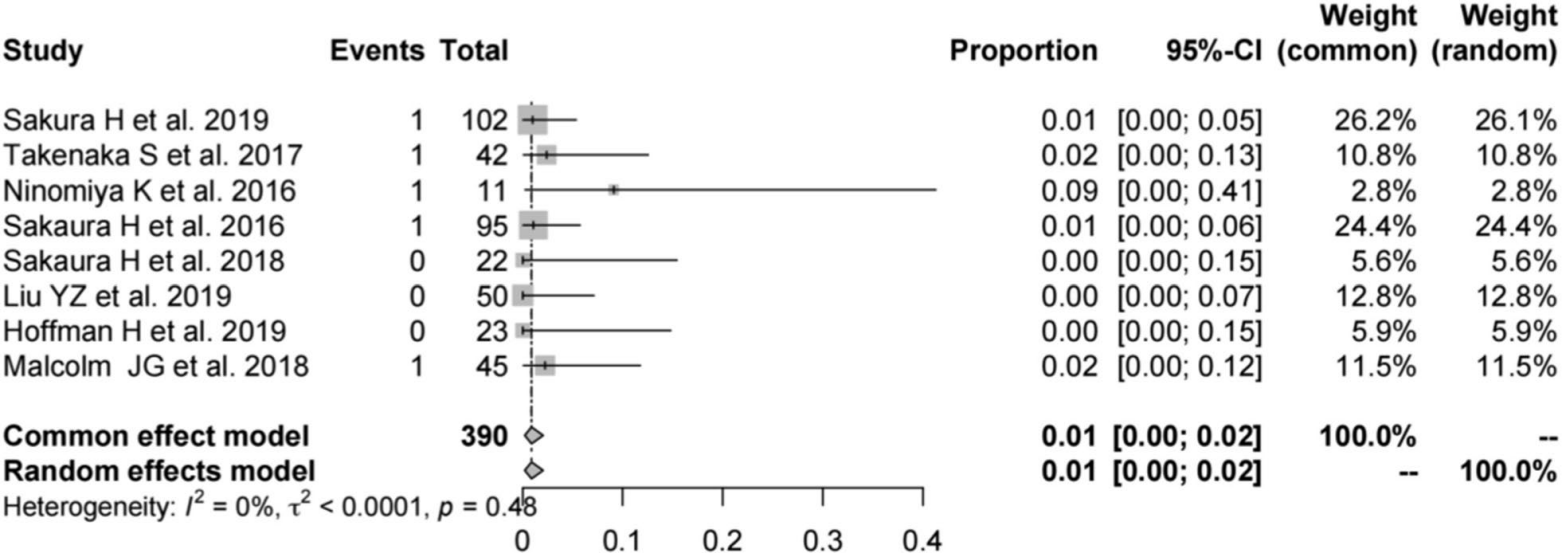
Meta-analysis of revision rate of CBT

Eight studies [14, 16, 19, 22, 23, 27, 37, 39] consisting of 390 patients reported the revision rate of CBT (*n* = 5). There was no significant heterogeneity (*I*^2^ = 0%, *P* = 0.48). Meta-analysis was performed using a fixed-effects model. Combined statistics showed a revision rate of 1% (95% CI [0, 2%]) (Fig. 17).

#### Revision rate of PS

**Fig. 18:**
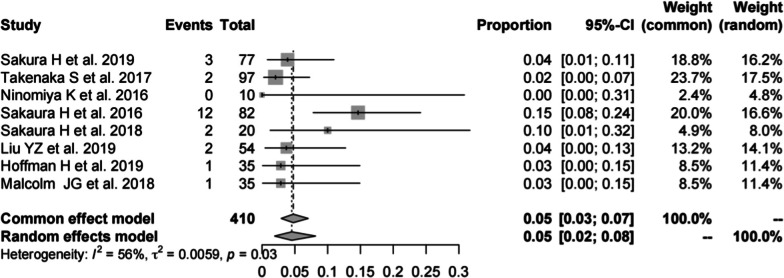
Meta-analysis of revision rate of PS

Eight studies [14, 16, 19, 22, 23, 27, 37, 39] (consistent with the revision rate of CBT) consisting of 410 patients reported the revision rate of PS (*n* = 23). There was a significant heterogeneity (*I*^2^ = 56%, *P* = 0.03). Meta-analysis was performed using a random-effects model. Combined statistics showed a revision rate of 5% (95% CI [2, 8%]) (Fig. 18).

### Sensitivity analysis

In this study, seven of the results showed *I*^2^ > 50%, including the total complication rate of CBT, total complication rate of PS, hardware complication rate of PS, ASD rate of PS, and revision rate of PS. The sensitivity analysis results were as follows (Additional file 1: Figure S1, Additional file 2: Figure S2, Additional file 3: Figure S3, Additional file 4: Figure S4, Additional file 5: Figure S5). Lai et al. [26] had a slight effect on the meta-analysis results of the total complication rate of CBT and PS, the other literature had no significant effect on the combined effect size, and the meta-analysis results were stable.

## Discussion

To solve the problem of decreased pedicle screw holding force caused by trabecular structure destruction and bone loss in patients with osteoporosis, which is leading to screw loosening and breakage, Santoni et al. [6] proposed the CBT technique in 2009. In recent years, the CBT technique has gradually become popular in clinical practice [11, 42]. However, there is still a lack of large-sample clinical studies, which affects the clinical application of the CBT technique.

At present, the studies on the clinical efficacy of CBT and PS techniques mainly focus on the comparison of complication rate, fusion rate, revision rate, operation time, blood loss, and other indicators. Keorochana et al. [43] and Qiu et al. [44] demonstrated that the CBT technique had a lower total complication rate than the PS technique. Wang et al. [8] demonstrated that the CBT technique had a lower ASD rate than the PS technique, but there were no significant differences in the hardware complication rate, wound infection rate, fusion rate, and revision rate. Kim et al. [45] confirmed that the CBT technique had lower ASD rate, total complication rate, and revision rate than the PS technique, but no significant differences in hardware complication rate, wound infection rate and fusion rate. Chang et al. [46] proved that the CBT technique had lower ASD rate and total complication rate than the PS technique, but no significant difference in hardware complication rate and wound infection rate. Zhang et al. [47] showed that the CBT technique had lower ASD rate and total complication rate than the PS technique, but there was no difference in fusion rate. Overall, the available studies concluded that the CBT technique was superior in reducing the incidence of ASD and total complications to the PS technique, but there were some controversies existed in fusion rate, revision rate, and hardware complication rate.

However, the aforementioned studies did not give specific statistics on complication rate, fusion rate, and revision rate for the two techniques with large sample sizes. This study synthesized 29 published studies to discuss the complication rate, fusion rate, and revision rate for the CBT and PS techniques in lumbar interbody fusion surgery. This study demonstrated that the total complication rate of the CBT technique was 6%, with a hardware complication rate of 2%, ASD rate of 1%, wound infection rate of 1%, dural damage rate of 1%, low hematoma rate tending to 0%, fusion rate of 94%, and revision rate of 1%. The total complication rate of the PS technique was 9%, with a hardware complication rate of 2%, ASD incidence of 3%, the wound infection rate of 2%, dural damage incidence of 1%, hematoma incidence tending to 0%, fusion rate of 94%, and revision rate of 5%. On balance, the results of this study are similar to those of published literature on the clinical efficacy of the CBT and PS techniques, further demonstrating the reliability of the findings of this study.

ASD was a common complication due to degeneration of the adjacent segmentafter lumbar interbody fusion surgery [48]. ASD occurs mainly in the upper segment above the fused segment and was considered to be an important factor affecting patient prognosis [48, 49]. The results of this study showed a slightly lower incidence rate of ASD with the CBT technique than with the PS technique, and we believe that this result is related to the following factors. First, soft tissue damage at the surgical site is a potential cause of ASD [49–52], and PS placement requires extensive expose of fascia, muscles, and ligaments, whereas the CBT technique requires only a small amount of muscle stripping at the paravertebral level to complete the surgery due to the inward insertion point, which causes less damage to soft tissues at the surgical site. Peng et al. [29] and Ohkawa et al. [53] measured blood creatine kinase concentrations in patients treated with the CBT technique and the PS technique respectively, and found that the blood creatine kinase concentrations in patients treated with the CBT technique were significantly lower than those in patients treated with the PS technique, which also indicated that the CBT technique caused less damage to the muscle tissue around the surgical site. Second, several biomechanical studies have shown that the increased disk stress in adjacent segments can lead to the development of ASD [49, 54, 55]. Liu et al. [56] demonstrated that the intervertebral disk stress in the adjacent segmentsfor pedicle screw fixation technique was significantly greater than that of the cortical bone trajectory technique in flexion, extension, lateral bending, and rotation, so the incidence of ASD was lower for the cortical bone trajectory technique than for the pedicle screw fixation technique. Third, degeneration of facet joints was an independent risk factor for the development of ASD [57, 58]. The PS placement inevitably damages the facet joint, which in turn leads to uneven forces on the upper lumbar disk during axial rotation and further decreases the stability of the vertebral body, thus further accelerating the development of ASD [59, 60].

Wound infections in lumbar interbody fusion surgery often lead to unfavorable prognoses [61]. The greater operative time, blood loss, postoperative drainage, and the size of the incision were the important risk factors for postoperative wound infection. Several studies [45–48, 62–65] demonstrated that the operative time, intraoperative blood loss, and the size of the incision of the CBT technique were significantly lower than those of the PS technique. In terms of postoperative drainage, Liu et al. [27] demonstrated that the postoperative drainage of the CBT technique (102 ± 10 ml) was significantly lower than that of the PS technique (246 ± 15 ml) and that the operative time, intraoperative blood loss, postoperative drainage, and the size of the incision were all superior to those of the PS technique, resulting in a lower rate of postoperative wound infection.

The conventional concept was that the revision rate of the CBT technique is higher than that of the PS technique because of the high difficulty of inserting the CBT screw. The CBT technique mainly passes through the cortical bone located at the lamina and the medial wall of the pedicle and has a higher cephalad angle and lateral angle, making it possible to cause the pedicle fracture or screw entering the spinal canal, which leads to a higher revision rate and a lower fusion rate [66, 67]. However, this study demonstrated that the revision rate of the CBT technique was slightly lower than that of the PS technique. The result is related to the following factors. First, screw malpositioning and loosening was the main cause of revision [66, 68], and some advanced techniques are often used as an aid when inserting the CBT screw, such as the use of a 3D printing guiding plate, intraoperative CT navigation, and robotics to confirm the entry point and trajectory, which can undoubtedly improve the accuracy of screw placement [69–71]. Also, due to the difficulty of the CBT technique, most of the surgeons have been systematically trained for increasing the accuracy of the procedure before operation. Second, cancellous bone is more prone to osteoporosis than cortical bone. For every 10 g/cm^3^ decrease in bone density, the maximum pullout resistance of the screw decreases by 60 N [72]. Compared with the PS technique, the CBT technique has a higher cephalad angle and lateral angle, and the vast majority of the screw trajectory was surrounded with the cortical bone, allowing for the better biomechanical stability [42, 73]. In this study, we concluded that the CBT technique has a higher fusion rate,lower revision and complication rate.

This study has several limitations. This study differs from the traditional meta-analysis, which can only yield superiority and inferiority between different techniques. This study is a single-arm meta-analysis to derive specific complication, fusion, and revision rate, based on which the two techniques were compared. Although this single-arm meta-analysis minimized the heterogeneity, some limitations remain. For example, to make the CBT technique comparable with the PS technique, only the literature that included both techniques was included, and those that used only one technique were not included. In addition, the studies included in this study were mainly retrospective studies, and there were only two RCTs, which may increase the bias. This study demonstrated that the total complication rate of the CBT technique was lower than that of the PS technique, in which there was only a slight difference in recent complications except for the rate of wound infection, but a large difference in remote complications. Further changes in complication,fusion and revision rate in the lumbar PS and CBT techniques need to be further evaluated by large-sample multi-center RCTs.

## Conclusion

The total rates of complications, ASD, wound infection, and revision of the CBT technique were lower than those of the PS technique in lumbar interbody fusion surgery, and there was a slight difference between the other results. CBT technique might be an alternative choice in clinical application.

## Supplementary Information

**Additional file 1: Figure S1.** Sensitivity analysis of total complication rate of CBT.

**Additional file 2: Figure S2.** Sensitivity analysis of total complication rate of PS.

**Additional file 3: Figure S3.** Sensitivity analysis of hardware complication rate of PS.

**Additional file 4: Figure S4.** Sensitivity analysis of the incidence of ASD in PS.

**Additional file 5: Figure S5.** Sensitivity analysis of revision rate of PS.

**Additional file 6:** **Table S1.** CBT and PS data summary.

## Data Availability

All datasets generated for this study are included in the article/Additional file 6.
