# Supplementary figures and images for: Complication, fusion, and revision rate in the lumbar cortical bone trajectory and pedicle screw fixation techniques: a systematic review and meta-analysis
Source: J Orthop Surg Res. 2023 May 25;18:382. doi: 10.1186/s13018-023-03820-7 (PMC10210483; doi:10.1186/s13018-023-03820-7)

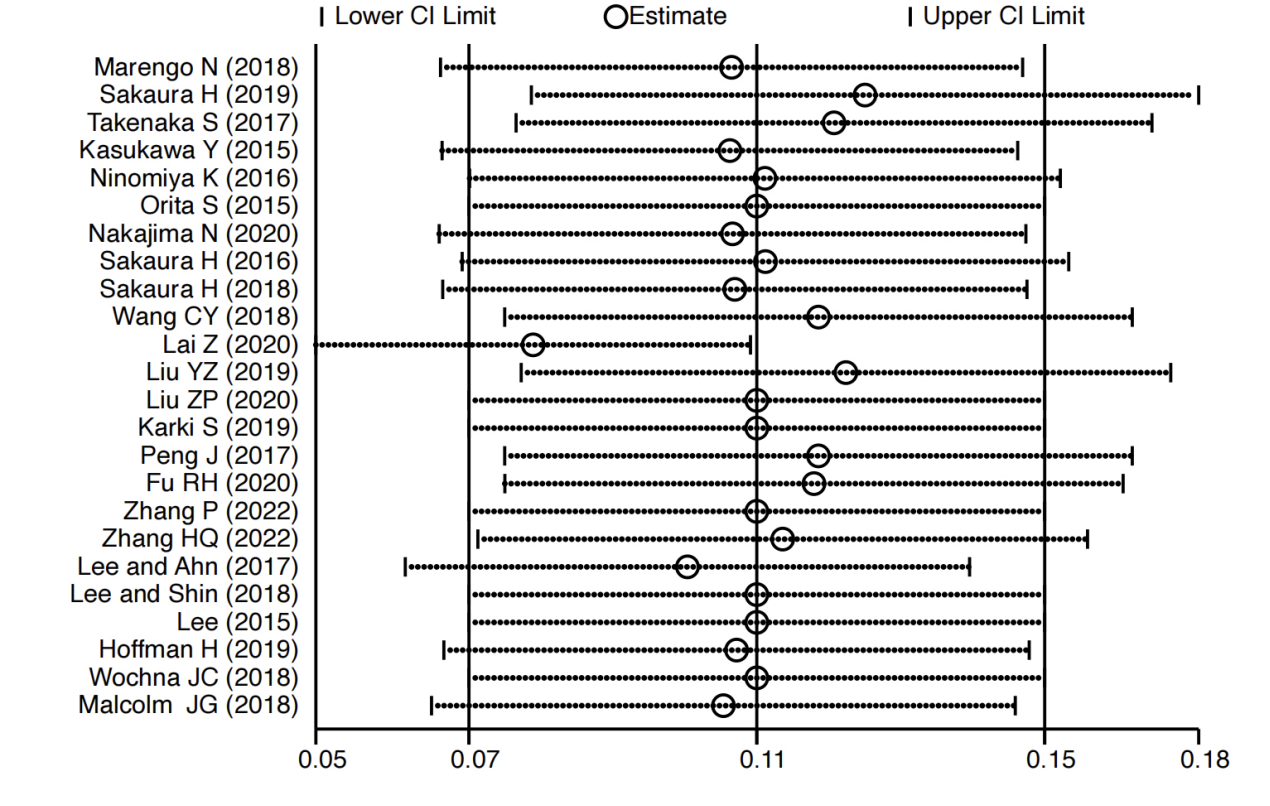

Supplement: Supplementary file 1 — Additional file 1: Figure S1. Sensitivity analysis of total complication rate of CBT. [file 13018_2023_3820_MOESM1_ESM.tif]

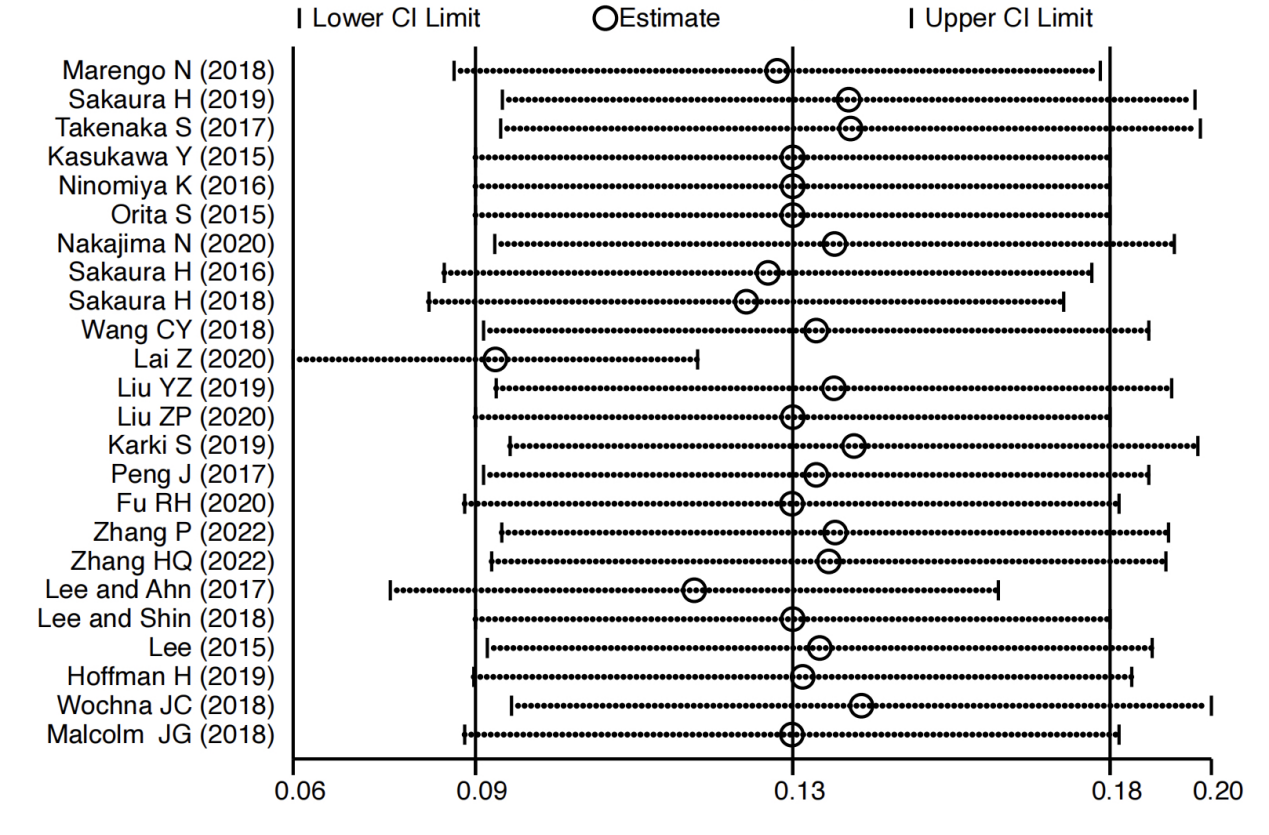

Supplement: Supplementary file 2 — Additional file 2: Figure S2. Sensitivity analysis of total complication rate of PS. [file 13018_2023_3820_MOESM2_ESM.tif]

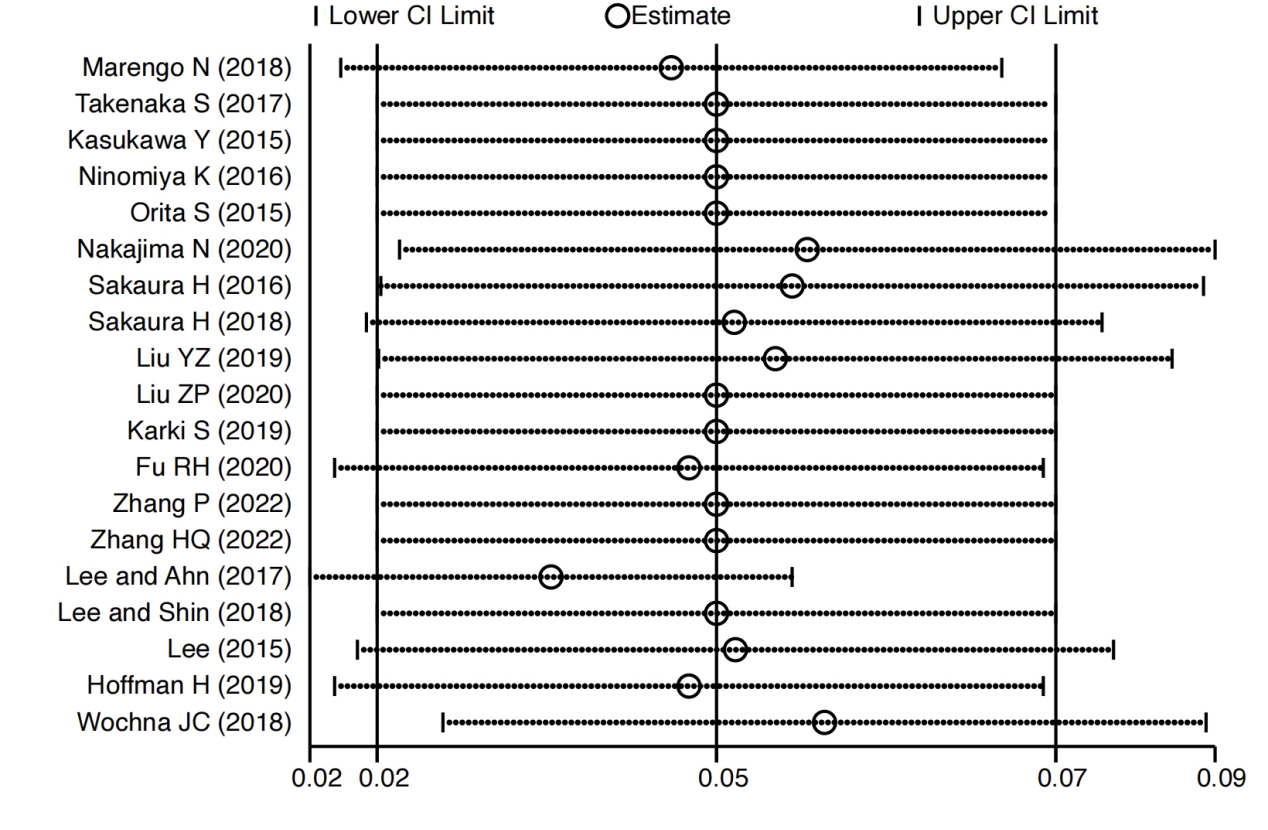

Supplement: Supplementary file 3 — Additional file 3: Figure S3. Sensitivity analysis of hardware complication rate of PS. [file 13018_2023_3820_MOESM3_ESM.tif]

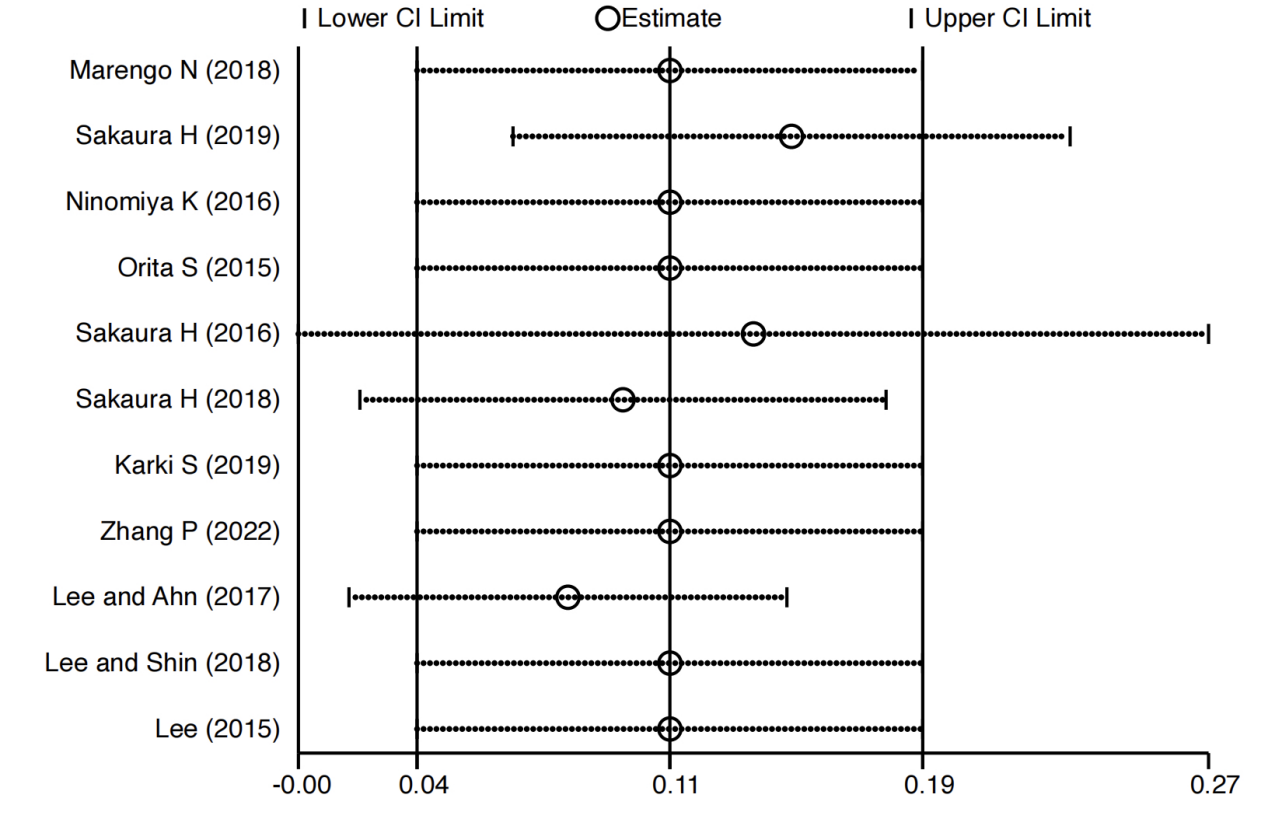

Supplement: Supplementary file 4 — Additional file 4: Figure S4. Sensitivity analysis of the incidence of ASD in PS. [file 13018_2023_3820_MOESM4_ESM.tif]

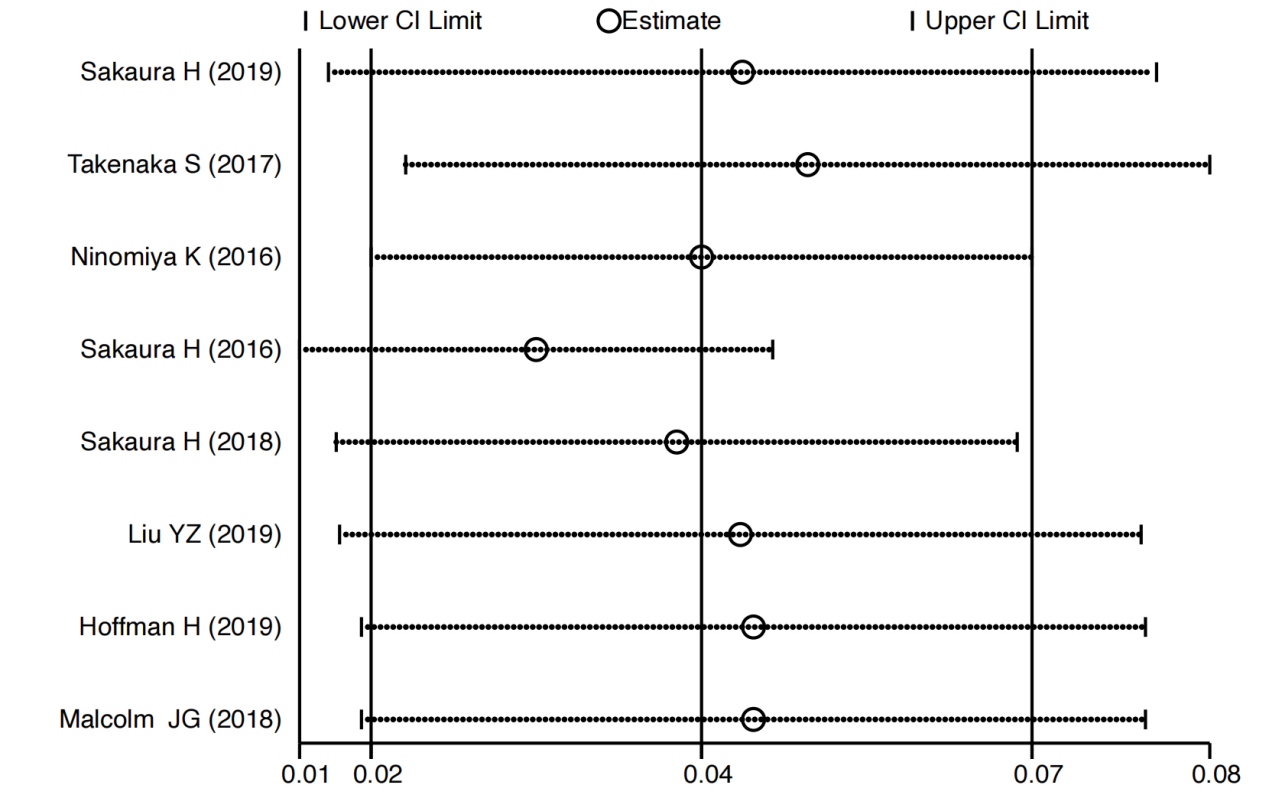

Supplement: Supplementary file 5 — Additional file 5: Figure S5. Sensitivity analysis of revision rate of PS. [file 13018_2023_3820_MOESM5_ESM.tif]
